# Management of radicular cyst associated with primary teeth using decompression: a retrospective study

**DOI:** 10.1186/s12903-022-02572-w

**Published:** 2022-12-01

**Authors:** Jun Pei, Shimin Zhao, Hui Chen, Jun Wang

**Affiliations:** 1 Department of Pediatric Dentistry, Shanghai Ninth People’s Hospital, Shanghai Jiao Tong University School of Medicine, Shanghai, China; 2 College of Stomatology, Shanghai Jiao Tong University, Shanghai, China; 3 National Center for Stomatology; National Clinical Research Center for Oral Diseases, Shanghai, China; 4 Shanghai Key Laboratory of Stomatology, Shanghai, China

**Keywords:** Primary teeth, Radicular cysts, Decompression, Space management

## Abstract

**Background:**

Radicular cysts arising from primary teeth are rare. Enucleation and marsupialization or decompression are treatment approach to odontogenic cysts. Decompression known to achieve good results in various cysts is widely used in clinic. This study aims to evaluate the efficiency of decompression in reducing radicular cysts associated with primary teeth in children.

**Methods:**

Cases of radicular cysts associated with primary teeth treated by decompression were reviewed in the present study. Clinical information and radiologic data of pre and post decompression were measured and analyzed.

**Results:**

Twenty-three patients treated for 25 cysts were included. All lesions with mean initial area 3.66 ± 2.00 cm^2^ were reduced after decompression time ranging 2 to 10 months. Mean rate of reduction was 0.77 ± 0.44 cm^2^/mo and large lesions (> 3.5 cm^2^) had a significantly higher reduction rate compared to smaller ones (< 3.5 cm^2^) (*P* < 0.00). All effected succedaneous teeth erupted after treatment at follow-up while 12 (46%) of them had root development problems.

**Conclusions:**

Decompression represents superiority as an effective and less invasive treatment in radicular cysts associated with primary teeth.

Trial registration: This study was retrospectively registered in the Ethics Committee of Ninth People’s Hospital Affiliated with Shanghai JiaoTong University School of Medicine (No.SH9H-2022-T158-1).

## Background

Radicular cyst is a most common type of odontogenic cyst which originates from epithelial cell rests of Malassez in periodontal ligament in response to inflammation of pulpal death and subsequent tissue necrosis [1]. However radicular cysts arising from primary teeth are rare, representing only 0.5%-3.3% of total number of radicular cysts both in primary and permanent dentitions [2]. The lesions are usually noticed by routine radiographic examination of primary teeth endodontic problems while some long standing lesions may cause appreciable expansion of the cortical bone and display clinical signs and symptoms like swelling, tooth mobility and displacement of an successional tooth [3].

Most studies consider that due to increased osmotic pressure within the lumen of the cyst, continuous hydrostatic pressure applying on peripheral bone makes radicular cysts enlarge [4–6]. Usually, there are two ways to conduct to treat odontogenic cysts – surgical enucleation or marsupialization, or a combination of the two techniques. Marsupialization, a large window created within the cyst bony, is a well-accepted and gradual diminishing technique for treatment of odontogenic cysts, as this forms a pouch connecting the oral and cystic cavities, allowing peripheral bone to fill the defect after relief of the internal hydrostatic pressure caused by the cystic content [7–10]. A technique termed decompression only requires a much smaller opening in the cystic wall to enable drainage of cystic content and loss of internal hydrostatic pressure, which is kept open usually by fixing a device (tube or stent) to its periphery [10, 11]. When clinical and radiographic features are suggestive of radicular cysts of primary teeth, extraction of the affected primary tooth is advised and decompression is a much preferred approach for children. The advantage of decompression reveals in preservation of unerupted successors, avoidance of surgical damage to closely adjacent anatomical structures, maintenance of oral tissues, minimal impairment of bone growth [11]. What’s more, mentally, children and parents are more likely to accept conservative and minimally invasive treatment.

There is a lack of relevant original research on the treatment of radicular cysts arising from primary teeth because of its low incidence. The objective of this retrospective study was to clarify the efficacy and the effectiveness of decompression as the preferred treatment of radicular cysts associated with primary teeth.

## Methods

### Data collection

A retrospective study was performed of children who were treated with decompression for radicular cysts associated with primary teeth. This study conforms Declaration of Helsinkiwas and approved by the Ethics Committee of Ninth People’s Hospital Affiliated with Shanghai JiaoTong University School of Medicine (No.SH9H-2022-T158-1). This study used the database of children who sought medical advice at the Department of Pediatric Dentistry, Ninth People’s Hospital Affiliated with Shanghai JiaoTong University School of Medicine. Patients were enrolled in the study if they met the inclusion criteria: 1) children were diagnosed as radicular cysts associated with primary teeth and treated with one uniform decompression approach; 2) with comprehensive clinical data and regular panoramic radiographs data.

**Fig. 1:**
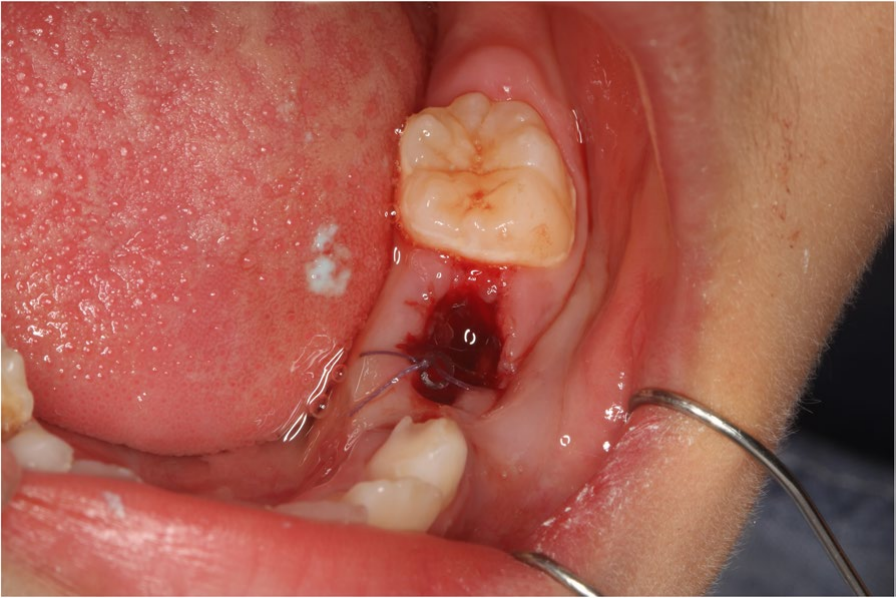
Decompression in one of the cysts. Tube placed immediately after tooth extraction during decompression

**Fig. 2:**
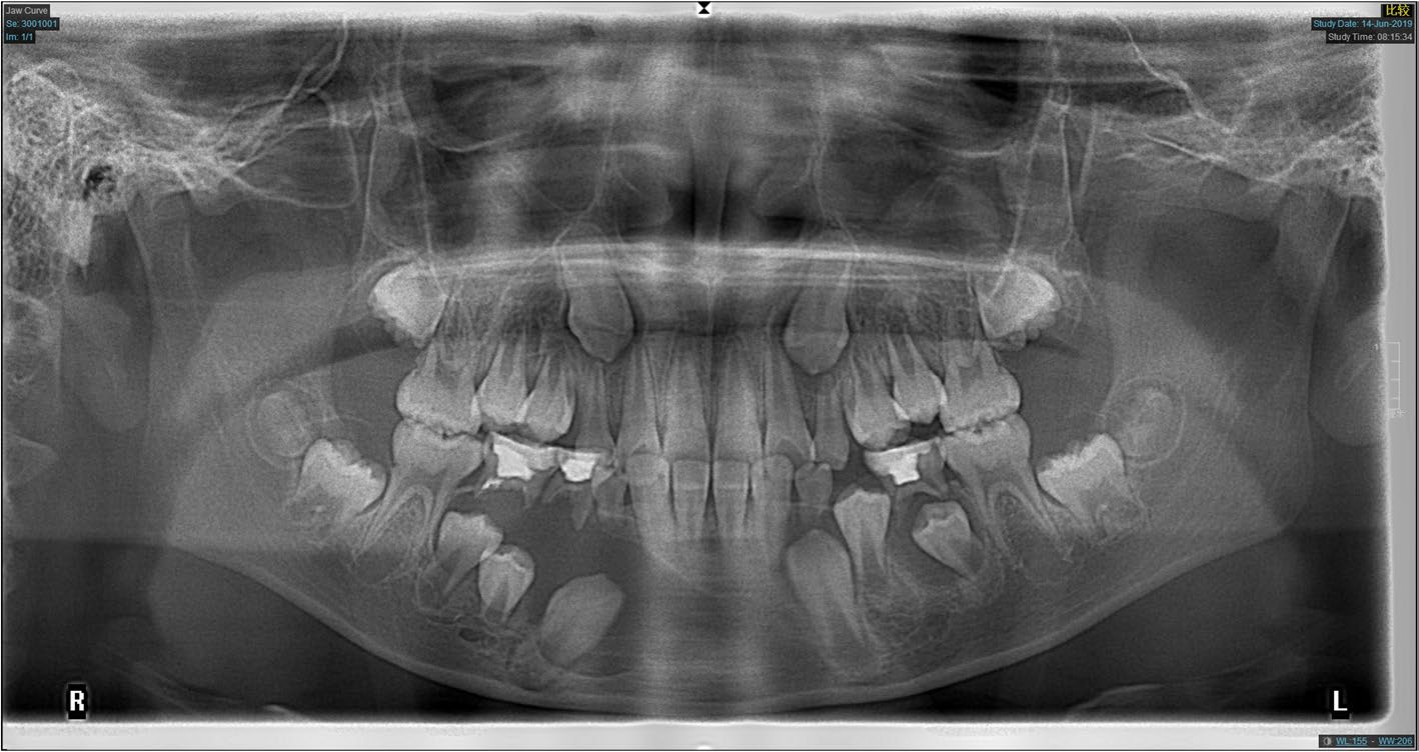
Panoramic radiograph of a 9-year-old boy at the first examination

**Fig. 3:**
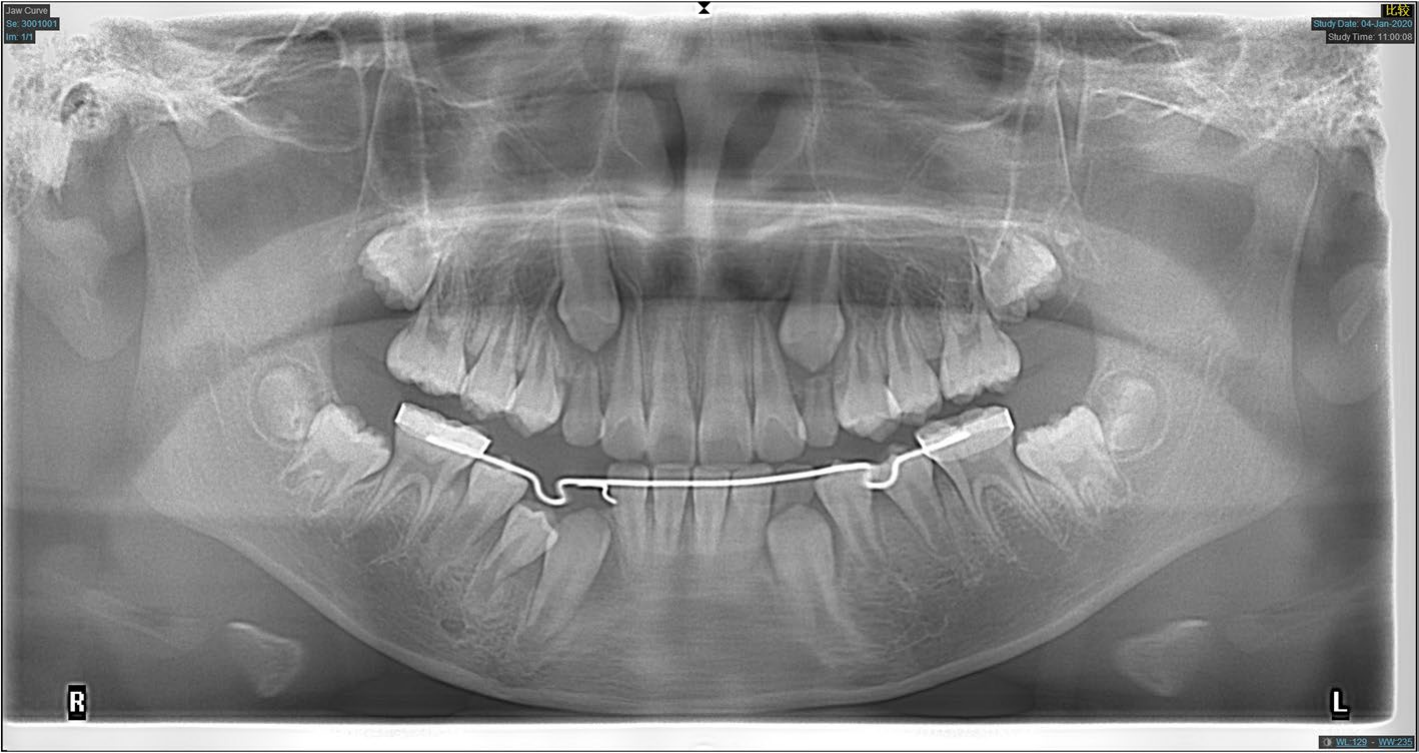
Panoramic radiograph of the patient shown in Fig. 2 after 6 months of decompression

Decompression protocol was as follows: patients were treated with extraction of the involved primary tooth. Tooth extraction socket generally connected with the cavity of cyst and cystic tissue was scraped for histopathologic examination to arrive at a final diagnosis. A resin tube fixed to gingiva by suture was placed inside of the alveolar hole to decompress the lesion. (Fig. 1) The resin tube was made by cutting off part of the pipe section from a new sterilized one-time indwelling needle. The surface of the tube needed to be brushed clean with a toothbrush every day after meals. A parent of the patient was instructed and asked to irrigate the lesion by a syringe with saline solution 0.9% every day. Regular follow-up was carried out once a month until the cyst diminished to disappear and the displaced or unerupted permanent tooth returned to normal eruption position. (Figs. 2 and 3) In the meantime, the necessary space maintainer would be performed.

Twenty-three patients were selected from December 2015 to March 2022. Data were collected including age, gender, presenting symptoms and signs, lesion location, primary tooth involvement, pre- treatment and post- treatment size, total decompression period, follow up of displaced or unerupted permanent tooth. All panoramic radiographs were obtained using OP/OC200 D digital panoramic/cephalometric x-ray. All evaluations were conducted by one examiner. Measurement of the irregular forms of radicular cysts was accomplished by the standard lesion area index (SLAI) as the maximal vertical length multiplied by the maximal horizontal length in centimeters defined by Anavi et al. [12]. Duration of decompression when the lesion disappeared basically was recorded. Monthly reduction rate (RR) was recorded as the SLAI divided by the decompression period in months. The condition about permanent tooth germs that were influenced by cysts also were recorded.

### Statistical analysis

Data were analyzed using SPSS Statistics, version 22.0 (IBM Corp., Armonk, NY, USA). The independent-samples t-test was applied to compare data of the variables between the groups and their monthly reduction rate. *P* values < 0.05 were considered statistically significant.

## Results

The study group consisted of 25 cyst lesions from 23 patients, 13 (57%) male and 10 (43%) female, of average age 7.9 years (range 5–11 years). The primary teeth that caused radicular cyst at presentation included 1 mandibular canine, 6 mandibular first molars and 19 mandibular second molars. There was 1 case which 2 adjacent problem teeth gave rise to one lesion. It can be seen that the second primary molar was the most involved tooth. All cases were in mandible. There were 9 cases that radicular cysts associated with primary teeth following pulp therapy. (Table 1).

**Table 1:** Descriptive data of the sample

Information	N	%		
Gender (*N* = 23 Patients)				
Male	13	57		
Female	10	43		
Involving primary teeth (*N* = 26 Teeth)				
Mandibular canines	1	4		
Mandibular first molars	6	23		
Mandibular second molars	19	73		
Pulp therapy (*N* = 26 Teeth)				
Yes	9	35		
No	17	65		

Radicular cysts were found most common when children were brought to seek medical advice because of swelling and fistula (17 [78%]). Few patients presented symptoms as tooth mobility (1 [4%]), swollen face (1 [4%]) and pain (1 [4%]). There were also 5 cases without any clinical presentation that were found during routine radiographic examinations. (Table 2).

**Table 2:** Presenting Signs and Symptoms (*N* = 25 Lesions)

Symptom	N	%
Swelling and Fistula	17	68
Swollen face	1	4
Tooth mobility	1	4
Pain	1	4
None (X-ray examination)	5	20

The mean initial SLAI was 3.66 ± 2.00 cm^2^ (range 0.37 to 7.09 cm^2^). After tooth extraction and decompression treatment, all cysts reduced markedly and completely disappeared when finished treatment. The mean treatment time of decompression was 4.96 ± 2.07 months (range 2 to 10 months) for all cases. The mean RR of all cysts was 0.77 ± 0.44 cm^2^/mo. (Table 3).

**Table 3:** Reduction rate of cysts by clinical parameters

Case	Initial SLAI (cm^2^)	Duration of Decompression (mo)	Reduction Rate (cm^2^/mo)
1	1.68	4	0.42
2	5.45	4	1.36
3	4.51	5	0.90
4	0.37	2	0.19
5	4.47	8	0.56
6	7.04	5	1.41
7	6.00	9	0.67
8.1	4.10	3	1.37
8.2	2.97	6	0.50
9	1.44	4	0.36
10	1.75	5	0.35
11	5.72	10	0.57
12	3.25	4	0.81
13.1	7.09	5	1.42
13.2	1.62	3	0.54
14	2.37	4	0.59
15	2.12	4	0.53
16	1.35	3	0.45
17	6.60	8	0.83
18	3.10	4	0.77
19	6.14	7	0.88
20	4.01	2	2.00
21	2.21	5	0.44
22	2.12	6	0.35
23	3.93	4	0.98
Average	3.66	4.96	0.77
SD	2.00	2.07	0.44

*Abbreviation*: *SD* Standard deviation

Distance between alveolar crest and the effected permanent tooth germ ranged 0.13 to 1.48 cm. 10 (38%) teeth germ still located on the normal eruption position, however 16 deviated (including the deviation of tooth germ as a whole and the rotation, transverse or inversion of tooth). In image when the cyst disappeared and the permanent tooth gradually erupted, 12 (46%) of the relevant permanent teeth had root development problems compared with teeth with the same name on opposite side. (Table 4).

**Table 4:** Relationship between permanent tooth germ and eruption position

Case	Distance from alveolar crest (cm)	Eruptive Position	Root development
1	0.40	normal	normal
2	0.25	normal	normal
3	0.83	normal	irregular
4	0.43	deviation	irregular
5	0.74	deviation	normal
6	1.00	deviation	irregular
7	0.62	deviation	irregular
8.1	0.54	normal	irregular
8.2	0.51	deviation	normal
9	0.51	deviation	irregular
10	0.13	normal	irregular
11	0.64	deviation	irregular
12	0.55	deviation	normal
13.1	0.94	deviation	irregular
13.2	0.42	normal	irregular
13.3	0.20	normal	normal
14	0.69	deviation	normal
15	0.18	normal	normal
16	0.45	deviation	normal
17	1.17	deviation	irregular
18	0.8	normal	normal
19	0.21	deviation	irregular
20	0.60	normal	normal
21	0.59	deviation	normal
22	0.16	deviation	normal
23	1.48	deviation	normal
Average	0.58		

The RR (Table 5) was similar in male and female. In this study, two groups were divided according to the size of largest cyst 7.09 cm^2^. Reduction occurred significantly faster in larger cysts, averaging 1.08 ± 0.43 cm^2^/mo in cysts with an initial SLAI of > 3.5 cm^2^ and 0.48 ± 0.17 cm^2^/mo in cysts with an initial SLAI of < 3.5 cm^2^ (*P* < 0.00).

**Table 5:** Reduction rate of lesions by clinical parameters

Parameter	N	Reduction rate (cm^2^/mo)	*P* value
Gender			
Male	15	0.76 ± 0.38	0.88
Female	10	0.79 ± 0.53	
Initial SLAI (cm^2^)			
< 3.5	13	0.48 ± 0.17	0.000
> 3.5	12	1.08 ± 0.43	

## Discussion

The results of this retrospective study illustrated that decompression was indeed an effective method for treatment of radicular cysts associated with primary teeth of low incidence.

In the study of Mass et al. [3], the primary molars were the teeth most often involved in radicular cysts in the primary dentition. Also, according to the study by Manekar et al. [13], the mandible was affected on cysts of deciduous molars more frequently than maxilla. In this study, all teeth involved were in mandible and 96% of them were molars which is consistent with the literature. The relationship of cyst development to the mandibular primary molars has been stressed as the teeth have a greater susceptibility to caries [14].

There seem specific clinical features of radicular cysts in association with pulpotomized primary molars: including buccal expansion, large size and rapid growth [15] 9 teeth (35%) in this study had received root canal therapy before developing cysts. This has also happened in previous reports. Mass et al. [3] reported 4 of 36 cases of cysts associated with primary molars following pulp therapy. In the study of Grundy et al. [15], there were 17 cases had received pulp therapy. Phenol group was the only composition common to the medicaments used in treatment. Grundy et al. estimated that antigenic necrotic materials in the root canals probably caused by pulpal therapeutic agents could provide continuing antigenic stimulation. This assumption does not imply that it is necessary to prohibit medicaments used for canal treatment of primary teeth, since a very low incidence of radicular cysts happens in primary teeth. In this regard, primary teeth after pulpal treatment should be followed up periodically.

Enucleation and marsupialization or decompression are two main treatments for odontogenic cysts. Even though enucleation can complete remove the lesion at one time, decompression a conservative approach has long been considered to achieve effective results in various types of odontogenic cysts [10, 11, 16–18]. Yoshikowa et al. [7] proposed a reduction of the intraluminal pressure in cysts made by decompression restores the original anatomy by the surrounding tissues, like bone and periost. Radicular cysts associated with primary molars are usually located in the periapical region and involve unerupted developing premolars of patients bellow 12 to 13 years old. The main advantage of decompression as a treatment for radicular cysts in children is obvious that it is less invasive and injury of permanent tooth buds could be avoided. Moreover, the study [11] in which the mean decompression time of patients < 18 years old was significantly less than that of adults implied osteogenic activity in children is higher.

Previous studies of decompression or marsupialization as treatment included various odontogenic cysts in all age groups. Nakamura et al. [16] performed marsupialization alone in 28 odontogenic lesions in an averaged 7.5 months and found 17% of cysts disappeared completely. Anavi et al. [11] reported 60% of cases were observed a good ossification of the area after decompression in odontogenic cysts, and 17 cases of radicular cysts had a slightly higher percentage of reduction of the lesion area (85.64%) than dentigerous cysts (81.52%) and keratocystic odontogenic cysts (78.85%). The finding of Gao et al. [19] also demonstrated that radicular cysts decreased more faster (3.37 cm^2^/month) than unicystic ameloblastomas (2.71 cm^2^/month) and keratocystic odontogenic cysts (2.87 cm^2^/month) at mean relative speed. Dror et al. [12] focused on decompression as a treatment of odontogenic cystic lesions in children and the mean percentage of reduction was 82%. The present study aimed to quantify the response of radicular cysts associated with primary teeth to decompression had a very effected result that all lesions disappeared completely in 4.96 ± 2.07 months.

In respect of factors influencing the decompression efficiency of cysts, Anavi et al. [11] and Asutay et al. [20] did not observe significant differences on gender, agreed with this study. In the present study statistically significant greater reduction rate occured in large cystic lesions than that in small ones after decompression. This is consistent with the previous studies [19, 21–25].

In this study succedaneous teeth were important to observed. In image of first visit, many permanent tooth germs were rotated or squeezed out of eruption position by cysts. After decompression treatment of radicular cysts all permanent tooth germs could erupt in study. In some cases, although permanent tooth germs were greatly affected by cyst s at first, they gradually developed normally and the prognosis was good at last. However, there were still 12 (46%) teeth that had root development problems when erupted, such as ectopic eruption, wide root canal, insufficient dentin deposition, insufficient root and root dilaceration. In this regard, the development of permanent teeth is worthy of long-term attention and follow-up after treatment for cysts. Early intervention and early treatment need prepared to prolong the survival time of permanent teeth.

Limitations of the study are mainly two points. Firstly, 23 patients in this study are still not enough to draw an exact conclusion but it can come to a hypothesis and tendencies. More cases of radicular cyst associated with primary teeth will be collected and analyzed continuously for future studies. Secondly, due to the ALARA principle, children did not take radiographic images every time they return, only if necessary, so the data cannot accurately represent the disease change of per time period.

## Conclusions

Decompression serves as an effective treatment in reducing radicular cystic lesions since it reserves the advantageous regeneration potential of the bone and soft tissues in the developing craniofacial skeleton of children and avoids injury to adjacent structures, which makes it more acceptable for children and parents in clinic.

## Data Availability

Data are available from the corresponding author upon reasonable request.
